# The Role of Chest Physiotherapy in Enhancing Quality of Life in a Postoperative Patient With Gingivobuccal Sulcus Carcinoma and Empyema: A Case Report

**DOI:** 10.7759/cureus.70492

**Published:** 2024-09-30

**Authors:** Muskan Qureshi, Lajwanti Lalwani, Samruddhi Aherrao

**Affiliations:** 1 Cardiorespiratory Physiotherapy, Ravi Nair Physiotherapy College, Datta Meghe Institute of Higher Education & Research, Wardha, IND

**Keywords:** active cycle of breathing techniques, chest physiotherapy, gingivobuccal sulcus carcinoma, numerating pain rating scale, short form 36

## Abstract

Gingivobuccal cancer encompasses a group of malignancies that affect the alveolus, retromolar trigone, buccal mucosa, and gingivobuccal sulcus (GBS). Among these, GBS carcinoma is the most prevalent malignant tumor found in the oral cavity. We present the case of a 56-year-old male who experienced pain in his right cheek for two months and was subsequently diagnosed with GBS cancer in the right buccal region, leading to surgical intervention. After one month, the patient was referred to chest physiotherapy due to complaints of cough with secretion and breathlessness. The chest physiotherapy protocol included deep breathing exercises, segmental breathing, and the Active Cycle of Breathing Technique, among others. Outcome measures used to assess progress included the Modified Medical Research Council Scale, Numeric Pain Rating Scale, 6-Minute Walk Test, and Short Form 36. The accompanying physiotherapy protocol demonstrated a positive effect on improving the patient’s quality of life, functional capacity, and exercise performance.

## Introduction

Oral cancer ranks 10th in incidence, contributing an additional 400,000 cases to the overall prevalence. Often referred to as the “Indian cancer,” gingivobuccal complex illness is the most common subsite in India and constitutes a significant portion of head and neck cancer cases across the Asian peninsula [1]. The extensive use of smokeless tobacco, particularly in northern states like Uttar Pradesh, Bihar, and Gujarat, remains the primary causative factor [1,2]. In Southeast Asia, oral cancer ranks third in prevalence [3]. In the Indian subcontinent, the gingivobuccal sulcus (GBS) is a prominent subsite for oral squamous cell carcinoma (SCC), and these tumors are known for their tendency to recur locally after treatment [4]. The increasing consumption of alcohol and tobacco significantly heightens the risk of developing this cancer [5].

Many patients often present as asymptomatic and are unaware of issues affecting their mandibular gingiva and buccal sulcus, leading to late presentations at advanced locoregional stages. Typically, locally advanced stages of mandibular GBS SCC involve soft tissue and bone degradation in the orofacial region, lymphatic node involvement in the neck, and potential remote metastasis. Radical resection and reconstruction of defects necessitate adjuvant treatments to achieve adequate locoregional control in these cases [6].

In instances of oral cancer, lower gingival tumors frequently present as T3 and T2 tumors, but T4 tumors are more common in invasive or advanced phases, characterized by lesions closely abutting the lower gingiva without radiological or clinical evidence of skeletal infiltration, indicating a marginal mandibulectomy [7]. The proximity of complex tumors to the mandible complicates surgical decisions. Some patients may consider mandibular conservation surgery an oncologically safe option, but segmental mandibulectomy is indicated in cases of extensive mandibular invasion or severe para-mandibular disease [8]. The rate of complications appears unaffected by the patient’s age, gender, or cancer location [9].

Common postoperative complications include restrictions in mouth movement, chest pain, dyspnea, edema, and limitations in activity near the surgical site [10]. The association of postoperative respiratory complications correlates with higher rates of mortality, unfavorable discharges to nursing homes, and increased healthcare utilization, such as longer hospital stays, unplanned admissions to intensive care or high-dependency units, and readmissions [11]. Clinical and radiological diagnoses of postoperative pulmonary complications, including pneumonia, bronchospasm, acute respiratory distress syndrome, and any degree or site of atelectasis related to pneumothorax and pleural effusion, were prospectively collected [12].

One well-known complication is pleural empyema, for which the best treatment involves drainage placement, prompt surgical intervention, and the administration of local antibiotics [13]. An empyema is defined as a collection of purulent material forming either from a gram-positive epithelium or culture obtained from the pleural fluid. This pathology most commonly occurs after thoracic surgery or trauma, as well as following pneumonia [14]. The aim of this study is to evaluate patient outcomes in chest physical therapy treatment concerning GBS cancer and to enhance the quality of life related to activities of daily living.

## Case presentation

Patient information

A 56-year-old male patient was referred to our hospital with complaints of pain in the right cheek and a nonhealing ulcer in the region of the right posterior jaw. Initially small, the ulcer had gradually enlarged to approximately 4 × 3 cm over the past two months. The patient reported experiencing a burning sensation after consuming hot and spicy foods for the same duration. Additionally, he mentioned applying a balm and undergoing hot fermentation 10-12 times in one month and three to four times in the subsequent month, respectively. The pain was characterized as dull, intermittent, and localized, exacerbated by mastication and relieved by rest and medication.

The patient had a history of consuming khara and tobacco three to four times daily for about 25 years, with no significant comorbidities such as diabetes, hypertension, bronchial asthma, or tuberculosis reported. An X-ray of the gingivobuccal cavity was performed, which did not support a provisional diagnosis. The patient was subsequently diagnosed with lower right GBS carcinoma and underwent surgery on March 6, 2024.

One month postoperatively, the patient presented with severe dyspnea, a productive cough with expectoration, chest discomfort during breathing, pain at the incision site, and episodes of intermittent fever. He was referred to the physiotherapy department for further management.

Written consent was obtained from the patient. He was conscious, cooperative, and oriented to time, place, and person. On assessment, the patient was positioned supine, with his head supported by a pillow. Both elbows and shoulders were in a neutral position, while his hips and knees were slightly flexed. The patient appeared ectomorphic in build. A Ryle’s tube, intravenous line, and intercostal drain (ICD) were in place. A pectoralis major myocutaneous flap had been used for reconstruction of the right jaw and neck.

The patient maintained adequate oxygen saturation in room air, with an abdominothoracic breathing pattern. His cough was noted to be effective. Upon palpation, chest expansion was reduced at the nipple and xiphoid levels, and tactile vocal fremitus in the left lung field showed dullness. The patient was hemodynamically stable, with all vital signs within normal limits. Auscultation revealed audible rhonchi over the left lung field.

Radiological findings

**Figure 1:**
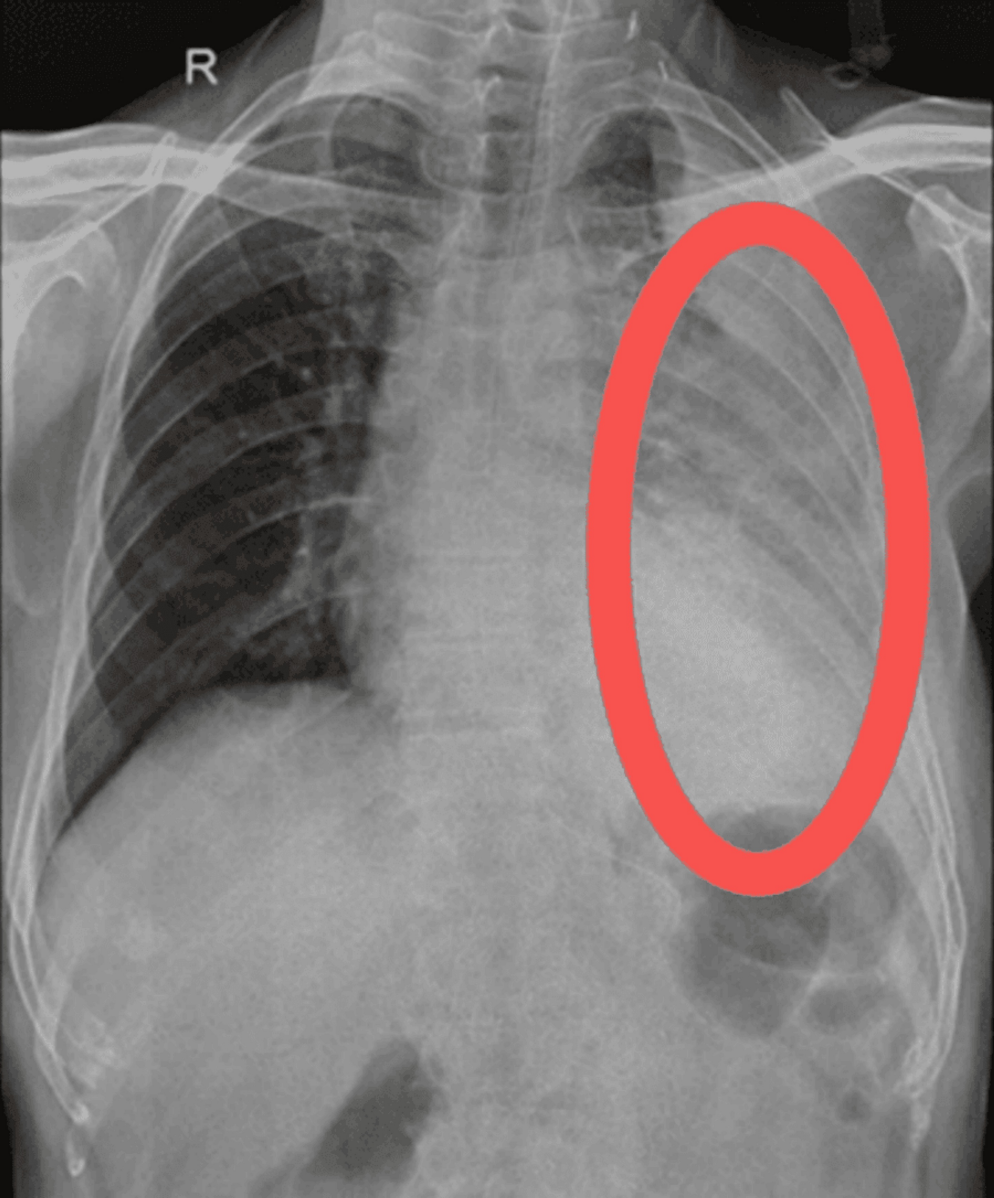
Pre-intervention chest X-ray showing consolidation in the upper and lower lobes of the left lung (marked with red oval)

**Figure 2:**
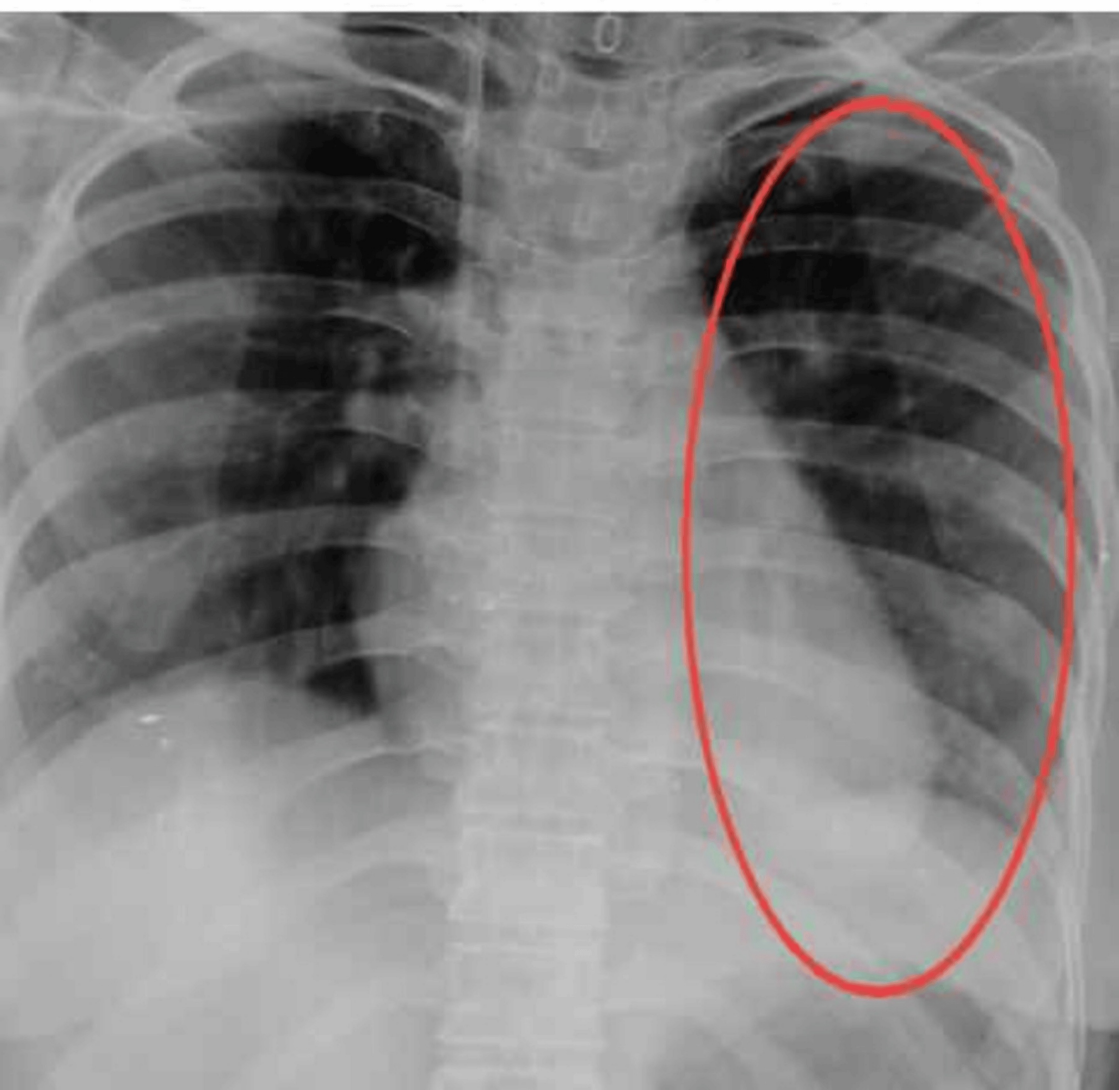
Post-intervention X-ray

An X-ray was performed, revealing consolidation in both the upper and lower lobes of the left lung. Additionally, a broad-based homogeneous opacity was observed in the left lower lobe. Figure 1 presents the pre-intervention X-ray, while Figure 2 displays the post-intervention X-ray.

Timeline of the events

Table 1 depicts the timeline of events.

**Table 1:** Timeline of events

Events	Timeline
Dates of admission	May 29, 2024
Date of surgery	June 3, 2024
Date of assessment	July 7, 2024
Date of physiotherapy rehabilitation	July 7, 2024
Date of post-assessment	July 19, 2024

Physiotherapy intervention

The physiotherapy interventions given to the patient are presented in Table 2.

**Table 2:** Physiotherapy intervention given to the patient FITT, frequency, intensity, time, and type; ICD, intercoastal drain

Problem list	Goals	Intervention	Dosage
Lack of knowledge	To educate the patient and his caregiver	Provide an explanation of the patient’s ailment and the physiotherapy treatment you want to initiate	
Accumulation of secretions	To remove and reduce the secretion	Active cycle of breathing technique	Two to three cycles during each session (two sessions per day)
Reduced aerobic capacity	To improve aerobic capacity	Circuit training and FITT principle. It assists in designing and structuring exercise programs. It includes aerobic exercises (spot marching and ambulation), strength training (resistive exercises for both upper and lower limbs), and flexibility exercises (shoulder flexion and chest expansion)	10 reps × 1 set (twice a day)
Reduced lung functioning	To improve lung functioning	Deep breathing exercises; segmental breathing exercises	Twice a day
Reduced peripheral muscle strength	To improve the strength of peripheral muscles	Strength training with 1 kg weight cuff for bilateral upper and lower limbs.	10 reps × 1 set (twice a day)
Pain at the incision site (ICD)	To reduce pain	Educate the patient about the splinting technique	
Improper posture	To improve the posture	Shoulder shrugs and rolls, scapular retraction, thoracic extension, and bilateral upper limb mobility	10 reps × 1 set (twice a day)
	Home exercises program to maintain the progression of intervention given	Breathing exercises, education of dyspnea relieving positions, upper limb and lower limb mobility exercises	10 reps × 1 set (twice a day)

Outcome measures

The Modified Medical Research Council (MMRC) Scale, quality of life, Numerical Pain Rating Scale, and 6-Minute Walk Test (6MWT) outcomes are presented in Table 3. 

**Table 3:** Outcome measures used in patients before and after treatment protocol 6MWT, 6-Minute Walk Test; ICD, intercoastal drain; MMRC, Modified Medical Research Council; NPRS, Numerical Pain Rating Scale; SF36, Short Form 36

Outcome	Pre-assessment	Post-assessment
MMRC Scale	Grade 3	Grade 1
Quality of Life (SF36)	42/100	58/100
NPRS (ICD)	6/10	2/10
6MWT	240 meters	315 meters

## Discussion

The gingivobuccal complex cancer approaches the buccal or labial gingiva by extending over the surface mucosa and the submucosal soft tissue. A marginal, segmental mandibulectomy is done for this case. Empyema refers to the collection of pus within a body cavity, usually in the pleural space, which goes on to complicate the rehabilitation process. Physiotherapy becomes even more important in such postoperative complications in patients with cancer developing empyema after surgery for carcinoma of the gingivobuccal region. It has been demonstrated that chest physical therapy is a successful strategy for enhancing the results. In this case, patients’ mental as well as physical health has been significantly improved as there is a notable increase in Short Form 36 (SF36) scores; this increase in SF36 scores highlights the crucial significance that physical therapy plays in the treatment of empyema by indicating a noteworthy enhancement in comprehensive health-related quality of life. MMRC scale for dyspnea has significantly improved after the protocol, showing better lung function and less respiratory discomfort after the treatment. The 6MWT distance showed a significant increase. The increment in the 6MWT distance is indicative evidence of substantial improvement in muscular strength, cardiovascular endurance, and total functional ability.

Mandhane et al. conducted a study on the quality of life in oral cancer patients post-mandibulectomy who had undergone physiotherapy protocol for two weeks, which included various breathing exercises. The study concluded that physiotherapy improved the patient’s quality of life and helped to prevent postoperative pulmonary issues [15]. Pathan et al. conducted a study on the physiotherapy approach toward reconditioning of patients with empyema that concluded breathing exercises help in improving lung function. Lung expansion techniques and an active cycle of breathing techniques led to decreased dyspnea in patients [16,17]. A randomized control trial conducted on oral cancer patients by Satish et al. stated that the 6MWT is closely linked to VO2 max, which makes it a reliable assessment tool for cancer patients [18]. Syed et al. conducted a crossover trial comparing the effect of the active cycle of breathing techniques and convectional chest physiotherapy in bronchiectasis patients in which the active cycle of breathing techniques was found to be effective [16]. Breathing exercises included pursed-lip, segmental, and diaphragmatic breathing, which improved ventilation by forcing airway back pressure [17]. A placebo-controlled trial was conducted in patients with chronic breathlessness by Currow et al., who used the MMRC scale in cardiorespiratory and palliative care services in Australia for his study, which was a randomized controlled trial [19]. This case report describes how chest physiotherapy played a vital role in improving the quality of life of a patient undergoing surgery for GBS cancer, followed by the resolving of empyema. Hence, physiotherapy input in the management of empyema in patients with gingivobuccal carcinoma is of essence toward the optimization of outcomes and undertaking a holistic approach to cancer care.

## Conclusions

This case report of a 56-year-old man with GBS carcinoma emphasizes the necessity of a multidisciplinary approach to treatment. The comprehensive rehabilitation program implemented for this patient, complicated by empyema, yielded significant improvements across multiple outcome measures. Notably, the Numerical Pain Rating Scale scores reflected a marked reduction in pain levels at the ICD site, enhancing comfort and facilitating greater participation in daily activities. The SF-36 scores demonstrated substantial gains in physical functioning, role limitations due to physical health, and overall health perceptions, indicative of an improved quality of life. Additionally, the patient’s MMRC dyspnea scale rating improved, revealing reduced shortness of breath during routine activities. This improvement was further substantiated by the 6MWT, which showed an increase in the distance covered, signifying enhanced functional endurance and cardiopulmonary capacity.

The findings from this case underscore the critical importance of a multidisciplinary approach in managing postoperative complications, particularly in complex cases such as GBS carcinoma with empyema. Early and comprehensive rehabilitation not only aids recovery but also empowers patients to regain functionality and enhance their overall well-being. Integrating physiotherapy into cancer care can significantly improve both physical and mental health outcomes, promoting a quicker return to normal daily activities and fostering a more positive outlook on life.
